# 3-Phenyllactic acid generated in medicinal plant extracts fermented with plant-derived lactic acid bacteria inhibits the biofilm synthesis of *Aggregatibacter actinomycetemcomitans*

**DOI:** 10.3389/fmicb.2022.991144

**Published:** 2022-09-23

**Authors:** Shrijana Shakya, Narandalai Danshiitsoodol, Masafumi Noda, Yusuke Inoue, Masanori Sugiyama

**Affiliations:** Department of Probiotic Science for Preventive Medicine, Graduate School of Biomedical and Health Sciences, Hiroshima University, Hiroshima, Japan

**Keywords:** 3-phenyllactic acid, *Aggregatibacter actinomycetemcomitans*, biofilm, lactic acid bacteria, fermentation, Paeonia Radix Alba, virulence

## Abstract

In the present study, the effect of PLA on a periodontic pathogen, *Aggregatibacter actinomycetemcomitans* (*A. actinomycetemcomitans*), the biofilm, and virulence-related genes was investigated. We confirmed that two lactic acid bacteria (LAB) strains isolated from plant sources, *Lactiplantibacillus plantarum* MSC-C2 and *Pediococcus pentosaceus* K40, secrete PLA into the de Man, Rogosa & Sharpe (MRS) broth when supplemented with phenyl pyruvic acid (PPA) as a precursor to PLA. Moreover, PLA was generated in the fermentation broths of two medicinal plant extracts, *Paeonia lactiflora* Pall (PR) and *Carthamus tinctorius* (CT), when used by each LAB strain and each extract supplemented with PPA. We determined that the minimum inhibitory concentration (MIC) of PLA against *A. actinomycetemcomitans* was 20 mM. PLA significantly decreased biofilm formation and suppressed the transcription of *pgA*, *ltxA*, and *cdtB* genes, which encode the poly-N-acetylglucosamine (PGA) polysaccharide of biofilm matrix and exotoxins leukotoxin and cytolethal distending toxin (CDT), respectively. The PLA produced by the MSC-C2 and K40 strains was increased several times by the addition of PPA to the MRS broth. The anti-biofilm effect of the extracts from the fermentation broth was proportional to the increasing PLA concentration, while a cumulatively higher effect than that of PLA alone suggested a combinational effect of PLA and the other metabolites, such as lactic acid (LA). Among the two medicinal plants, PLA, produced after the addition of PPA, was higher in PR extract in case of both the LAB strains. PLA production by the MSC-C2 strain in the PR extract reached 4.8 ± 0.23 mM, which was obviously higher than that in the MRS broth (3.88 ± 0.12 mM) supplemented with 1 mg/ml PPA. The activity to inhibit biofilm formation in the fermented PR extract was clearly high. PLA formed in the fermented PR extract downregulated the dispersin B encoding the *dspB* gene together with *pgA*, *ltxA*, and *cdtB*. In conclusion, this study shows a promising activity of PLA against the *A. actinomycetemcomitans* biofilm and virulence genes. In addition, the combinational effect of PLA and the medicinal plant extract can be achieved by fermentation with a specific plant-derived LAB strain.

## Introduction

An organic acid, 3-phenyllactic acid (2-hydroxy-3-phenylpropanoic acid or β-phenyllactic acid; PLA), is useful for food biopreservation due to its broad-spectrum antimicrobial activity (Rajanikar et al., 2021). PLA exhibits effective antagonistic activity against pathogenic bacteria such as *Listeria monocytogenes* (Dieuleveux and Gueguen, 1998), *Salmonella enterica* (Zhou et al., 2020), *Escherichia coli* O157:H7 and *Staphylococcus aureus* (Ohhira et al., 2004), and *Enterococcus faecalis* (Wang et al., 2018). The compound is also effective against fungi such as *Candida* and *Rhodotorula* species (Schwenninger et al., 2008) as well as Fusarium, *Aspergillus*, and *Penicillium* (Lavermicocca et al., 2003). PLA is associated with multifaceted antibacterial mechanisms of action, such as damaging the cell membrane and intercalating DNA (Ning et al., 2017). There are also emerging reports on the anti-biofilm activity of PLA against *Enterobacter cloacae* (Liu et al., 2018), *Pseudomonas aeruginosa* (Chatterjee et al., 2017), *Listeria monocytogenes* (Liu et al., 2017), and *Enterococcus faecalis* (Liu et al., 2020). Thus, PLA has potential as an antimicrobial and anti-biofilm candidate against other pathogenic microorganisms.

PLA, which is detected in honey (Tuberoso et al., 2011), is known to be produced by *Geotrichum candidum* (Dieuleveux and Gueguen, 1998) and *Bacillus coagulans* (Zheng et al., 2011). Some PLA-producing lactic acid bacteria (LAB), such as *Lactobacillus*, *Enterococcus*, *Leuconostoc*, and *Pediococcus* (Valerio, 2004; Zhang et al., 2014), have been also reported. Since they are generally regarded as safe (GRAS) microorganisms, PLA-producing LAB strains are gaining wide attention (Rajanikar et al., 2021). For PLA biosynthesis in LAB, the most important biochemical pathway is reported to be the amino acid metabolism, which involves the transamination of phenylalanine to phenyl pyruvic acid (PPA) and the subsequent PPA reduction to PLA by lactate dehydrogenase (LDH; Li et al., 2008). Since PLA is produced in natural and fermented foods in low amounts, De Man Rogosa Sharpe (MRS) broth or dairy whey broth with an additional supplementation of phenylalanine or PPA has been widely used to increase production yields (Valerio, 2004; Li et al., 2007; Rajanikar et al., 2021).

*Aggregatibacter actinomycetemcomitans*, a Gram-negative bacterium, colonizes the human oral cavity and is often associated with periodontal disease and sometimes with non-oral infections, including endocarditis (Zambon, 1985). It is thought to be a community activist in dysbiotic polymicrobial community–induced periodontitis and a producer of major virulent factors, such as exotoxins leukotoxin and cytolethal distending toxin (CDT), associated with the suppression of host defenses (Fine et al., 2019). It forms dental biofilm matrices, majorly comprising poly-b-1,6-N-acetyl-D-glucosamine (PGA), a hexosamine-containing polysaccharide that mediates intercellular adhesion to colonize the oral cavity (Izano et al., 2008). *Aggregatibacter actinomycetemcomitans*, which forms an oral biofilm, has shown higher resistance and is not easily phagocytized by immune cells; therefore, the conventional management of this pathogen-associated periodontitis with antibiotics has been difficult (Jaffar et al., 2016).

The aim of this study is to evaluate the potential of PLA against the biofilm and virulence genes of the periodontopathogen *A. actinomycetemcomitans*. Our research group has isolated over 1,200 strains of plant-derived LAB from fruits, vegetables, flowers, and medicinal plants and evaluated their health benefits, such as immune modulation, liver function improvement, and reduction of obesity (Jin et al., 2010; Zhao et al., 2012; Noda et al., 2018). Further, when cultivated in plant extracts, some of the plant-derived LAB-produced substances that have shown therapeutic potential (Okamoto et al., 2020; Shakya et al., 2021). Therefore, we also determined the PLA production by *L. plantarum* MSC-C2 isolated from sugarcane and *P. pentosaceus* K40 isolated from banana in extracts from the medicinal plants *Paeonia lactiflora* Pall and Carthamus tinctorius and studied the potential of PLA-containing LAB-medicinal plant extracts against the biofilm and virulence genes of periodontopathogen *A. actinomycetemcomitans*.

## Materials and methods

### Strains and culture conditions

*Aggregatibacter actinomycetemcomitans* ATCC 29523 (serotype a) was grown in Brain Heart Infusion broth (Merck, Germany) with 1% yeast extract (BHI/YE) under static and anaerobic incubation at 37°C for 24 h. LAB strains were grown in MRS broth (Merck, Germany) at 37°C for 24 h. For PLA production in MRS broth, the *L. plantarum* MSC-C2 K40 and *P. pentosaceus* K40 strains, which were isolated from sugarcane and banana, respectively, were grown in MRS broth alone or MRS broth supplemented with PPA (Sigma, United States) at 1 mg/ml or 3 mg/ml at 37°C for 24 h.

For the fermentation of medicinal herb extracts, the dried root of *Paeonia lactiflora* Pall (PR) or *Paeonia Radix Alba* and the dried flower of *Carthamus tinctorius* (CT) or Safflower were purchased from Kojima Kampo Co., Ltd. (Japan), and aqueous extracts were prepared at 1% *w/v* concentration. After filtration with a 0.22 μm membrane filter (Advantec Ltd., Japan), the cells from overnight cultures of the LAB strains were suspended in PR or CT extracts with or without 1 mg/ml PPA and incubated for 24 h at 37°C for fermentation.

### Preparation of PLA-containing LAB extracts

Since LA is a major organic acid produced by LAB strains, we compared the effects of PLA and LA on *A. actinomycetemcomitans* cultured for 24 h. The standard compounds of PLA and LA (Wako, Japan) were used for the biofilm inhibition test against *A. actinomycetemcomitans* at concentrations of 5, 10, 15, 20, and 25 mm. These concentrations were chosen based on the MIC of PLA against other bacteria as reported in previous studies (Fang et al., 2022). Meanwhile, PLA-containing LAB extracts were prepared from 24-h fermentation of the LAB cultures in MRS broth, PR extract, or CT extract with or without PPA, by centrifugation at 8,000× *g* for 10 min to obtain the respective cell-free supernatants (CFSs). After membrane filtration, the PLA-containing extracts from each CFSs were prepared by extracting three volumes of ethyl acetate and drying the upper ethyl acetate layer with a vacuum desiccator. These dried extracts were redissolved in two volumes of BHI/YE broth and were used for biofilm inhibition test against *A. actinomycetemcomitans*.

### Determination of PLA and LA concentrations

PLA concentration was measured after redissolving the above-mentioned dried ethyl acetate extract in an equal volume of methanol. Then a 10 μl aliquot of the extract was subjected to HPLC (JASCO system; JASCO Corporation, Japan) with a Crestpak C18S (5 μm, 30 nm, Φ = 4.6 mm, L = 150 cm) column (JASCO Corporation, Japan), eluted with a mobile phase of 35% methanol containing 0.05% trifluoroacetic acid (TFA) for 8 min at a flow rate of 1 ml/min, and detected at a wavelength of 209 nm. The PLA concentration (mM) was determined by curve fitting with a PLA standard (Wako, Japan).

The lactic acid (LA) concentration was determined by enzymatic reaction of the extracts with L-lactate dehydrogenase (L-LDH) and D-lactate dehydrogenase (D-LDH) in the presence of a glycine buffer and nicotinamide adenine dinucleotide (NAD) at 37°C for 30 min, and the absorbance was measured at 340 nm.

### Determination of percentage reduction of growth and biofilm of *Aggregatibacter actinomycetemcomitans*

Optical density (OD) of 24-h test samples-treated bacterial cultures at 595 nm and a previously described crystal violet staining method with slight modifications (de Canha et al., 2020) were used for the determination of percentage (%) reduction of growth and biofilm of *A. actinomycetemcomitans*, respectively. Briefly, in a 96-well plate, the 24-h culture of *A. actinomycetemcomitans* was suspended at a 2% *v/v* concentration into the fresh BHI/YE broth with or without PLA or LA at concentrations of 5, 10, 15, 20, and 25 mm. In the later experiment, the bacterial culture at 2% *v/v* concentration was suspended in the PLA containing LAB extracts prepared as mentioned earlier. They were incubated statically for 24 h in anaerobic condition. The OD of the planktonic cell growth was measured at 595 nm, for the determination of % reduction of bacterial growth. The concentrations of PLA and LA that inhibited more than 95% of the cell growth were considered as the minimum inhibitory concentration (MIC). Then, for the determination of % reduction in 24-h biofilm formation, the media were removed from the same plates, and were gently washed with 100 μl of distilled water three times. Adhered cells were then fixed using 100 μl of methanol for 20 min. After aspirating the methanol, the plates were dried and treated with 100 μl of 0.5% crystal violet for 20 min. The plates were then gently rinsed with distilled water to remove the excess crystal violet and allowed to air dry. The bound crystal violet was then dissolved using 100 μl of a 33% acetic acid solution, and absorbance was measured at 595 nm.

For the disruption of preformed biofilms, *A. actinomycetemcomitans* was plated as described above. After 24 h of adhesion, the media were removed, and the plates were gently washed with distilled water to remove planktonic cells. Following the washing step, 100 μl of the test sample in BHI/YE broth was added, the plates were incubated for an additional 24 h, and the biofilm was quantified using crystal violet.

The effect of test samples against *A. actinomycetemcomitans* 24-h cell growth, 24-h biofilm formation, and the preformed biofilm was calculated as a percentage reduction in OD at 595 nm as compared to the control sample, i.e., BHI/YE media without any test sample, as follows:

% Reduction = (OD_control_ − OD_test sample_)/OD _control_ × 100.

### Total RNA extraction and quantification of *Aggregatibacter actinomycetemcomitans* gene expression

The transcription of biofilm- and virulence-related genes of *A. actinomycetemcomitans* was determined by RT-qPCR. After treatment of 24-h bacterial culture with various concentrations of PLA or LA or PLA-containing LAB extracts for 4 h, the cell pellet was collected by centrifugation at 8,000× *g* for 10 min. Then, the total RNA was isolated using a NucleoSpin RNA Plus (Macherey-Nagel GmbH & Co. KG, Germany) and reverse-transcribed using the ReverTra Ace qPCR RT Master Mix with gDNA Remover (Toyobo, Japan) according to each manufacturer’s instruction manual. Real-time PCR consisted of 0.4 μmol of each primer, a KAPA SYBR Fast qPCR Kit (Kapa Biosystems, United States), and cDNA (4 ng/μl) and was conducted on the PikoReal Real-Time PCR System (Thermo Fisher Scientific, United States). The primers used in this experiment are as follows: 5′- ACGCTGTAAACGGTGTCG-3′ and 5′- TTGCATCGAATTAAACCACAT-3′ for 16 s rRNA, 5′- GACGGTGATGCGGTATTGG-3′ and 5′- GACCGATGATGGAGCTGAA-3′ for *pgA*, 5′- CAACAACACAATTCCAACCC-3′ and 5′- GGCGATACCTGTCCATTCTT-3′ for *cdtB*, 5′- ATCAGCCCTTTGTCTTTCCTAG-3′ and 5′- TGACCAAGTAAACTATCGCCG-3′ for *ltxA*, and 5′- ATACCATCAGCCTTTCCGGC-3′ and 5′- GGCATTTTCCGCACGTTGAT-3′ for *dspB*. Reactions were performed with an activation step of denaturation at 95°C for 10 min, amplification, and 40 cycles of 95°C for 15 s and 60°C for 1 min. Ct differences between the target gene in test and control samples were normalized to the reference gene 16 s rRNA Ct values and adjusted according to the efficiency of the reaction (Ishikawa et al., 2021).

### Statistical analysis

Data were expressed as the mean ± SD from at least three independent experiments. The significance of differences was determined *via* one-way ANOVA, followed by a *post hoc* Tukey test, and differences with *p* < 0.05 were considered statistically significant.

## Results

### Effect of PLA against *Aggregatibacter actinomycetemcomitans* in comparison with LA

**Figure 1:**
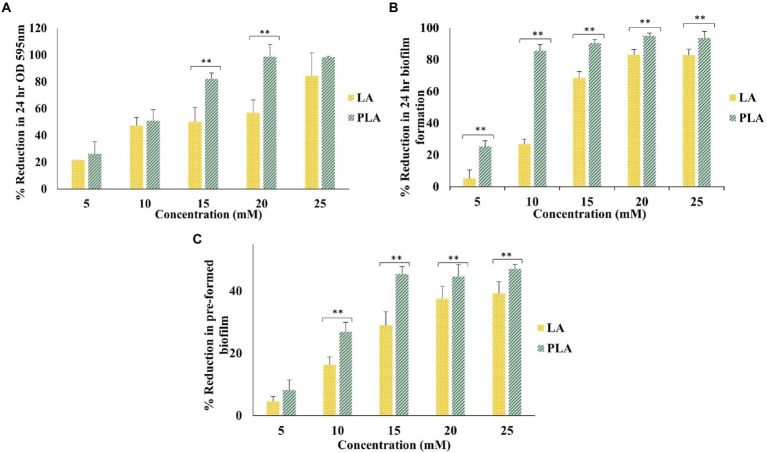
Effects of LA and PLA against *Aggregatibacter actinomycetemcomitans*: **(A)** 24 h growth as measured by OD at 595 nm, **(B)** 24 h biofilm formation, and **(C)** 24 h preformed biofilm, expressed in terms of percentage (%) reduction as compared to the control (BHI/YE media without test sample). The concentrations of LA and PLA were 5 mM, 10 mM, 15 mM, 20 mM, and 25 mM. Data represent the mean value from three independent assays in triplicate, and error bars represent the standard deviation (SD). Differences between groups were determined *via* one-way ANOVA. **p < 0.05 between the groups represented in the figure.

Since LA is a major organic acid produced by LAB strains, we compared the antagonistic effects of PLA and LA on *A. actinomycetemcomitans* cultured for 24 h, for the formation of biofilm, at concentrations of 5, 10, 15, 20, and 25 mm. As shown in Figure 1, concentration-dependent reductions were observed in the 24-h growth and biofilm formation by *A. actinomycetemcomitans* in the presence of PLA or LA. It was found that the MIC that inhibited more than 95% of the cell growth as measured by OD at 595 nm was 20 mm for PLA, while it was more than 25 mm for LA. At lower concentrations, i.e., 5 and 10 mm, both LA and PLA reduced the 24 h OD by around 20 and 50%, respectively, without any significant differences, while at 15 and 20 mm concentrations, the reduction by PLA was significantly higher than that by LA. Interestingly, it was found that even at 5 and 10 mm, there were significantly higher biofilm reductions by PLA than by LA, i.e., LA reduced the biofilm by only about 5 and 27% at these concentrations, while PLA succeeded to reduce it by 25 and 85%, respectively. In fact, PLA inhibited biofilm formation significantly in all tested concentrations as compared to LA. PLA decreased the biofilm formation by more than 90% at a sub-inhibitory concentration of 15 mm, while LA could only decrease it by 82% at 25 mm. In the case of preformed *A. actinomycetemcomitans* biofilms, although there was a significantly higher reduction by PLA than LA at 10–25 mm, both PLA and LA could only show reductions of less than 50% in all the tested concentrations. These data indicated that PLA is superior to LA in inhibiting cell growth and biofilm formation as well as in disrupting the preformed biofilm of this periodontopathogen. Moreover, among these inhibitory activities, the anti-biofilm activity of PLA was found to be particularly prominent, and these data suggested that mechanisms other than decreasing the planktonic cell growth or disrupting the biofilm matrices could be involved.

### Effect of PLA against *Aggregatibacter actinomycetemcomitans* genes

**Figure 2:**
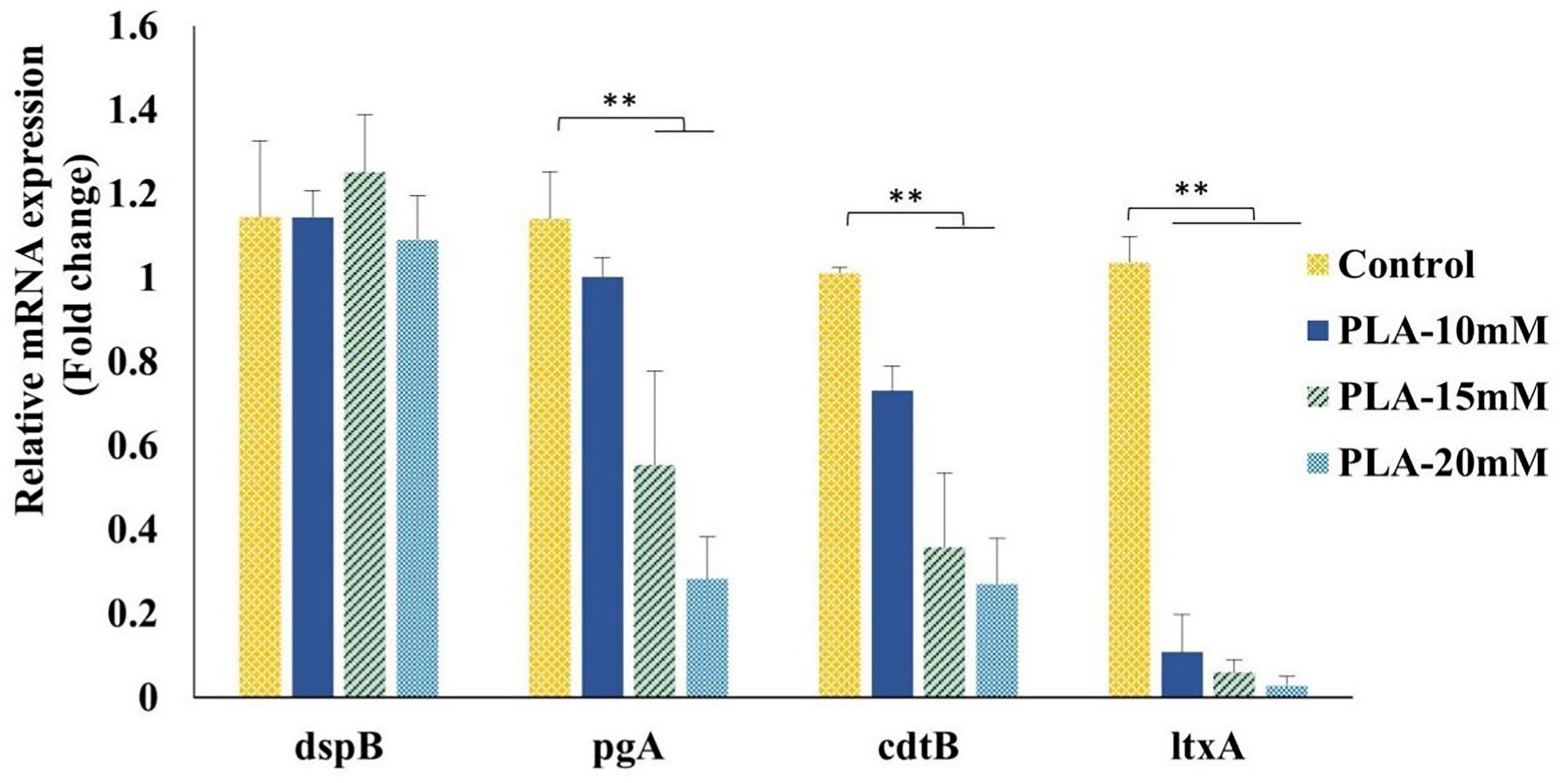
Effects of PLA on *A. actinomycetemcomitans* gene expressions when used at 10, 15, and 20 mM. Total mRNA was isolated, cDNA levels were determined by RT-qPCR, and Ct values were normalized to 16sRNA data. Data are expressed in terms of fold changes relative to the control (BHI/YE media without a test sample). Data represent the mean value from three independent assays in triplicate, and error bars represent the standard deviation (SD). Differences between groups were determined *via* one-way ANOVA. **p < 0.05 between the groups represented in the figure.

The potential activity of PLA against biofilm formation by *A. actinomycetemcomitans* led us to investigate its effect on transcription of the biofilm- and virulence-related genes. We determined the expression profiles of two biofilm-related genes, *dspB* and *pgA*, encoding dispersin B and the biofilm matrix polysaccharide PGA, respectively, along with the two major virulent toxins, leukotoxin and CDT, encoded by *ltxA* and *cdtB*, respectively, in *A. actinomycetemcomitans* when treated with 10, 15, and 20 mm PLA, as shown in Figure 2. It was found that PLA at any tested concentration did not modulate the expression of dispersin B; however, PLA was found to downregulate the expression of *pgA* concentration dependently and significantly at 15 and 20 mM. On the other hand, both virulence-associated genes, *ltxA* and *cdtB*, were also markedly suppressed by PLA in the increasing order of its concentration. The gene *ltxA* was found to be particularly downregulated by PLA, more than 10-fold. These results pointed toward the role of PLA in interfering with biofilm matrix polysaccharide synthesis, leading to anti-biofilm formation, as well as suppressing the virulence-associated genes of *A. actinomycetemcomitans*.

### PLA production by LAB strains

**Figure 3:**
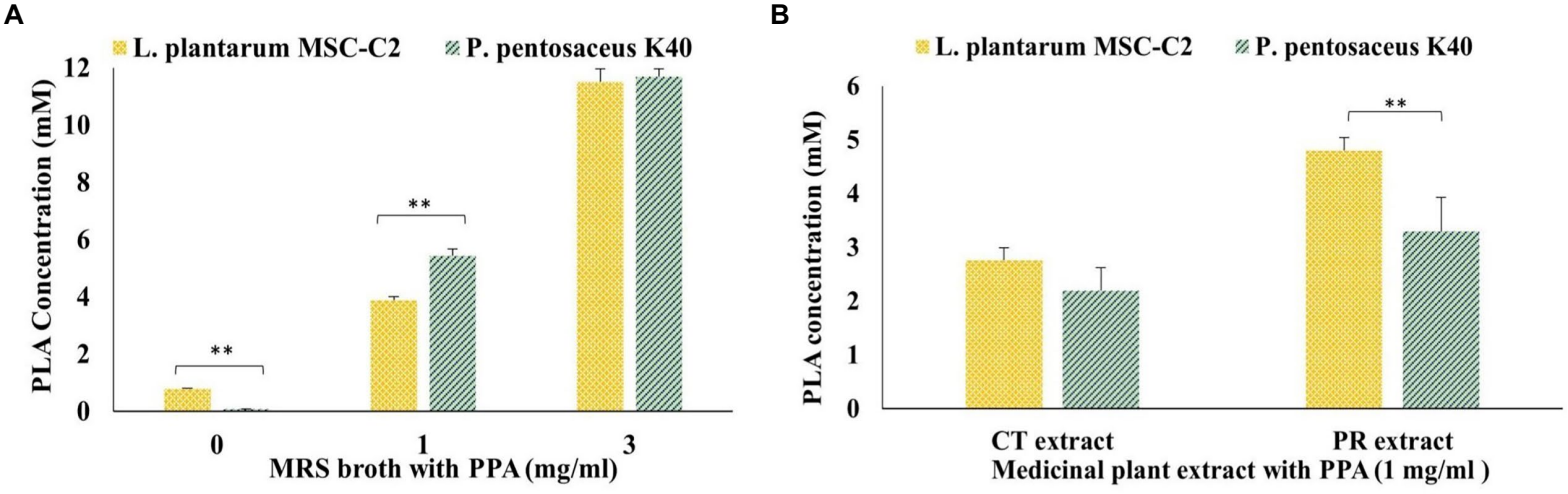
Concentration of PLA produced by MSC-C2 and K40 **(A)**, in MRS broth supplemented with or without PPA and **(B)**, in the PR extract and CT extract supplemented with PPA (1 mg/ml). The PLA concentrations were measured *via* HPLC after ethyl acetate extraction from the CFS and determined using a standard PLA concentration curve. Data represent the mean value from three independent assays in triplicate, and error bars represent the standard deviation (SD). Differences between groups were determined *via* one-way ANOVA. **p < 0.05 between the groups represented in the figure.

Owing to the remarkable activity of PLA against *A. actinomycetemcomitans* biofilm and virulence genes, we determined the PLA concentrations produced by MSC-C2 and K40 strains in MRS broth alone or with supplementation of 1 mg/ml and 3 mg/ml PPA, as depicted in Figure 3A. MSC-C2 had the ability to produce PLA as high as 0.8 ± 0.012 mm, while K40 only produced 0.08 mm of PLA in MRS broth without any supplementation at 37°C for 24 h. Since PPA is the direct precursor of PLA, the PLA production by both strains was greatly increased after PPA supplementation in MRS broth. Surprisingly, after adding 1 mg/ml PPA to the MRS broth, there was a 67.8-fold increase in the PLA produced by strain K40, but PLA increased by only 4.85-fold in strain MSC-C2, reaching 3.88 ± 0.125 mm and 5.43 ± 0.24 mm in MSC-C2 and K40 broths, respectively. However, when PPA at 3 mg/ml was added, the PLA concentrations reached about 11 mm in both strains. Thus, significant differences in PLA production between the two strains were found in MRS broth alone and MRS broth with 1 mg/ml PPA, but none with MRS broth containing 3 mg/ml PPA.

Furthermore, as shown in Figure 3B, PLA production by MSC-C2 and K40 was also observed in two medicinal plant extracts, *Paeonia Radix Alba* (PR) and *Carthamus tinctorius* (CT), when supplemented with 1 mg/ml PPA and fermented at 37°C for 24 h. Among the strains, MSC-C2 produced the highest PLA amount, i.e., 4.8 ± 0.23 mM in the PR extract, which was even higher than it could produce in MRS broth with 1 mg/ml PPA. In the same PR extract, strain K40 produced a significantly lower amount of PLA, i.e., 3.3 ± 0.63 mM. Meanwhile, both strains were able to produce relatively low concentrations of PLA in the CT extract, in the range of 2.2–2.76 mM. These results demonstrated that medicinal plant extracts like the PR extract with PPA could also be suitable for PLA production by some LAB strains.

### Effect of PLA-containing LAB extracts on the *Aggregatibacter actinomycetemcomitans* biofilm

**Figure 4:**
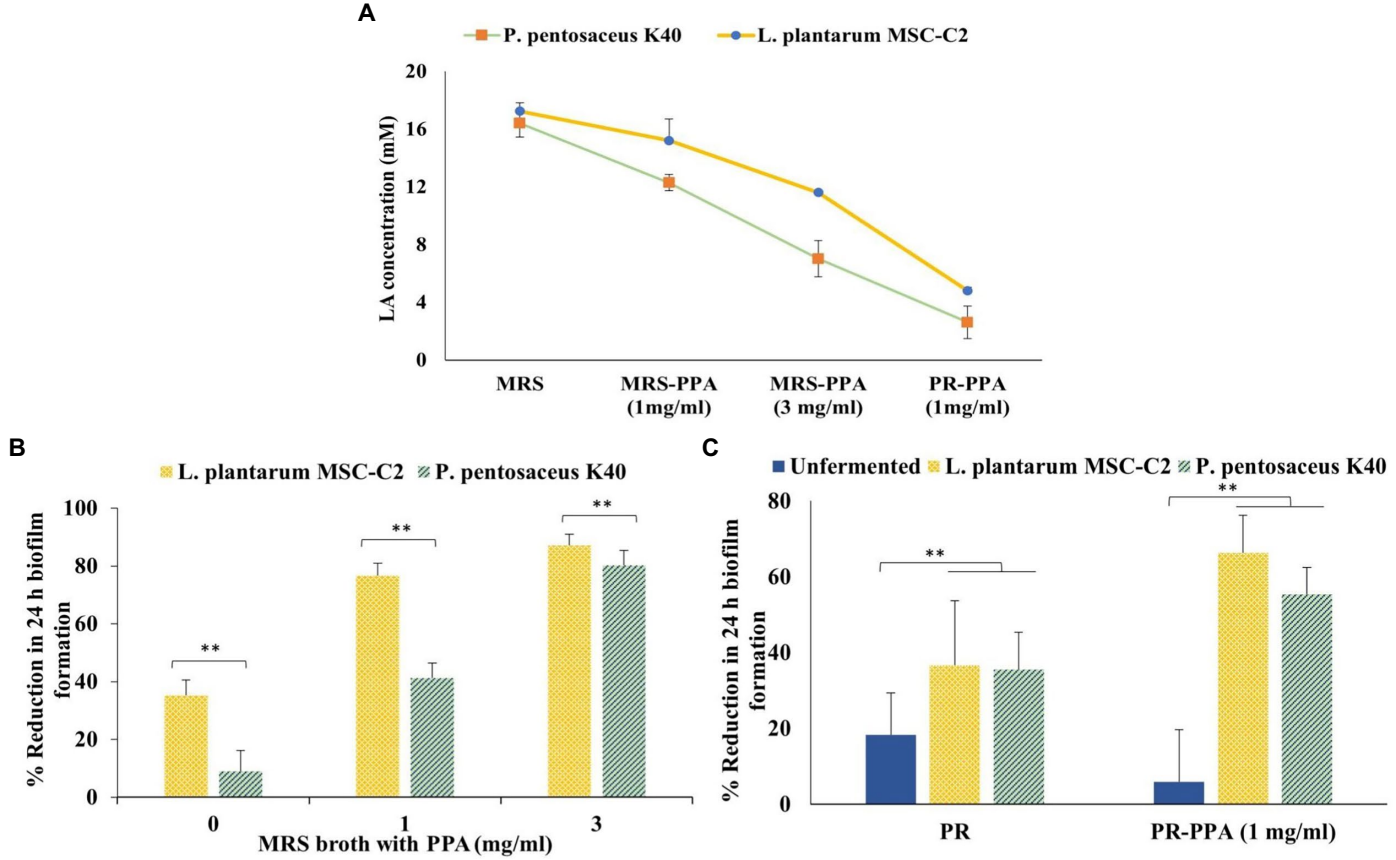
**(A)** Concentration (mM) of LA in PLA-containing LAB extracts from MRS broth, MRS-PPA (1 mg/ml), MRS-PPA (3 mg/ml), and PR-PPA (1 mg/ml). **(B)** Effect of PLA-containing LAB extracts against 24 h biofilm formation by *A. actinomycetemcomitans* from MRS broth, MRS-PPA (1 mg/ml), and MRS-PPA (3 mg/ml). **(C)** Unfermented and LAB-fermented PR extract with or without 1 mg/ml PPA. Data represent the mean value from three independent assays in triplicate, and error bars represent the standard deviation (SD). Differences between groups were determined *via* one-way ANOVA. **p < 0.05 between the groups represented in the figure.

Next, we determined the effect of fermented extracts of K40 and MSC-C2 strains from MRS broth or PR extract on biofilm reduction. As LA is the major organic acid produced by the LAB, during these experiments, the amount of LA extracted along with PLA was taken into consideration, and the LA concentration in these extracts was determined as shown in Figure 4A. In the case of MRS broth, the extracted LA concentration was detected to decrease with the addition of PPA and subsequent increase in PLA concentration, such that the concentration ranged from 16 to 17 mm and 12 to 15 mm in MRS broth alone and MRS broth supplemented with 1 mg/ml PPA, respectively. The LA concentration was decreased to 7 and 11 mm in the K40 and MSC-C2 strains, respectively, when the added PPA was increased to 3 mg/ml. Meanwhile, the LA concentration was lower in fermented PR extracts, such as 2.62 mM and 4.8 mM in the K40 and MSC-C2 strains, respectively.

In the subsequent experiment, we observed that the effect of PLA generated in MRS broth on the biofilm formed by *A. actinomycetemcomitans* increased when PPA was supplemented to each K40 and MSC-C2 strain, as shown in Figure 4B. All broths fermented by the MSC-C2 strain decreased the biofilm formation more strongly than their respective K40 extract counterparts. Due to the lower amount of PLA produced by the K40 strain than the MSC-C2 strain in MRS broth, there was more than a 3-fold difference in biofilm reduction between these two extracts. It was also noted that the effects of these extracts were higher than those of the respective concentrations of PLA alone. Hence, in these extracts, the LA concentration plays a synergistic role with PLA in inhibiting the biofilm formed by *A. actinomycetemcomitans*.

As shown in Figure 4C, the inhibitory effects on biofilm formation of unfermented and fermented PR or PR-PPA extract fermented with each K40 and MSC-C2 strain were determined. We observed that the medicinal plant PR extract, without fermentation, had a slight effect on the *A. actinomycetemcomitans* biofilm formation. This effect was significantly increased from approximately 18 to 35% after fermentation with either of the LAB strains. Noteworthily, the PR extract with 1 mg/ml PPA supplementation drastically increased the reduction percentage of biofilms to 55 and 66% after fermentation with K40 and MSC-C2, respectively. This marked increase could be attributed largely to the PLA concentration, as LA was produced in low amounts in these extracts. As shown in Figure 1B, PLA alone at a concentration of 5 mM produced about a 25% reduction in biofilm formation, the even lower PLA concentration in the PLA containing fermented PR extracts could also hint at a possible synergism between PLA and the PR extract after fermentation.

### Effect of PLA-containing LAB-PR extract against the virulence-gene expressions of *Aggregatibacter actinomycetemcomitans*

**Figure 5:**
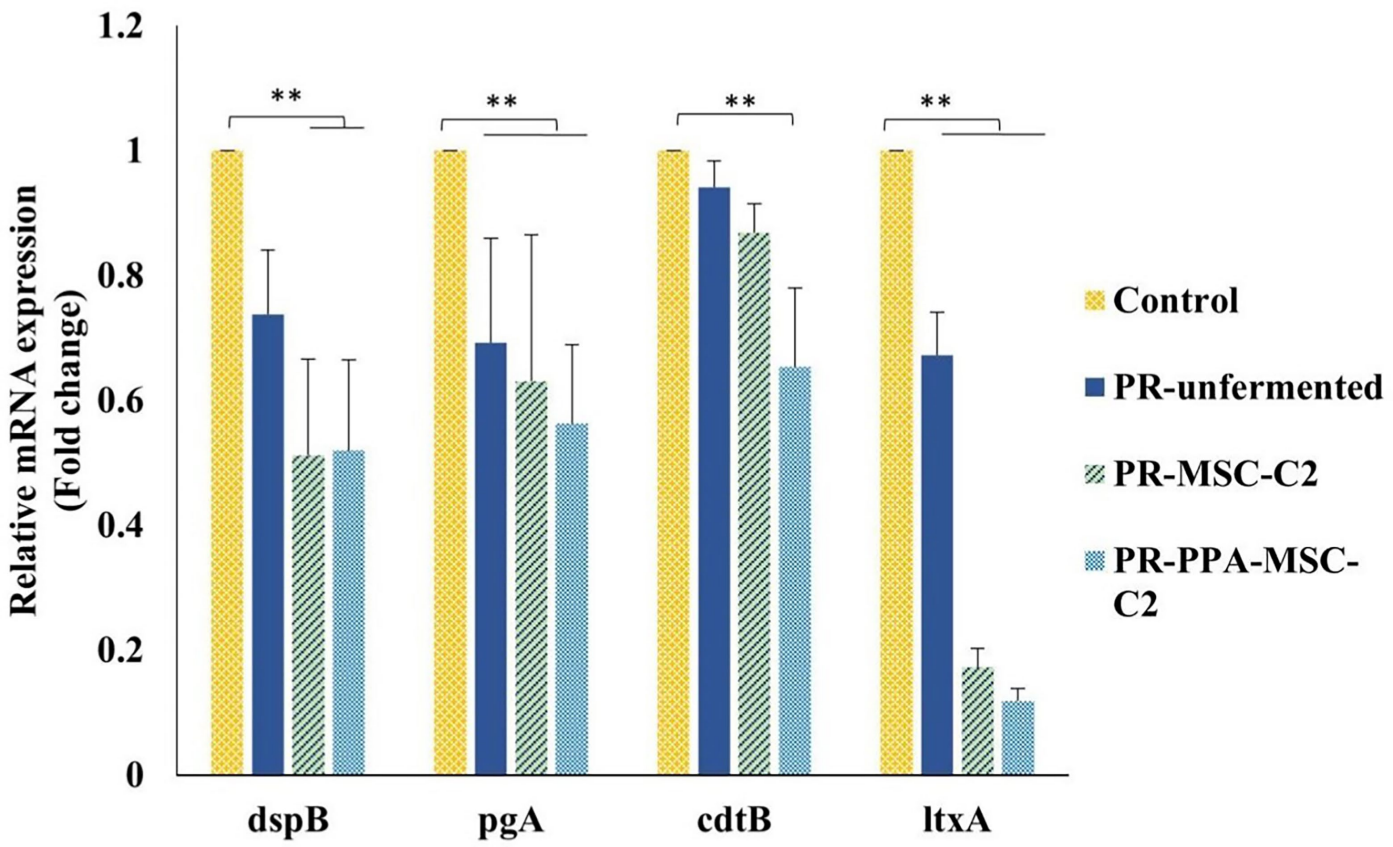
Effects of PLA-containing LAB-PR extract against *A. actinomycetemcomitans* gene expressions. Control, PR-unfermented, PR-MS-C2, and PR-PPA-MSC-C2 represent the BHI/YE without a test sample, unfermented PR extract, MSC-C2 fermented PR extract, and MSC-C2 fermented PR extract with 1 mg/ml PPA, respectively. Total mRNA was isolated, cDNA levels were determined by RT-qPCR, and Ct values were normalized to 16sRNA data. Data are expressed in terms of fold changes relative to the control (BHI/YE media without a test sample). Data represent the mean value from three independent assays in triplicate, and error bars represent the standard deviation (SD). Differences between groups were determined *via* one-way ANOVA. **p < 0.05 between the groups represented in the figure.

Finally, the effects of the unfermented PR extract, MSC-C2-fermented PR extract, and MSC-C2-fermented PR extract with 1 mg/ml PPA against the *A. actinomycetemcomitans* virulence genes were investigated. As shown in Figure 5, it was observed that the fermented PR extract downregulated all four target genes when compared with the unfermented PR extract. The fermented PR extract without PPA significantly repressed the transcriptions of *dspB*, *pgA*, and *ltxA* genes. However, the expression of *cdtB* was only decreased with the addition of PPA.

## Discussion

PLA, which is a normal metabolite produced by many LAB strains, exhibits a broad antimicrobial spectrum (Rajanikar et al., 2021). The compound is effective against numerous Gram-positive bacteria, such as *Listeria monocytogenes* (Zhang et al., 2022), *Staphylococcus aureus* (Ohhira et al., 2004), *Bacillus cereus* (Ning et al., 2021), *Enterococcus faecalis* (Wang et al., 2018), and *Enterobacter cloacae* (Liu et al., 2018), and Gram-negative bacteria like *Escherichia coli* (Ning et al., 2017), *Salmonella* (Zhou et al., 2020), *Shigella flexneri* (Jiang et al., 2022), *Klebsiella oxytoca* (Liu et al., 2021a), *Vibrio parahaemolyticus* (Fang et al., 2022), and *Pseudomonas aeruginosa* (Chatterjee et al., 2017).

In this study, we demonstrated the antagonistic effect of PLA on a periodontal pathogen, *A. actinomycetemcomitans*, with an MIC of 20 mM or 3.3 mg/ml, as shown in Figure 1A. Another research team has reported that the MICs of PLA against other bacteria ranged from 1.5 to 5.0 mg/ml (Fang et al., 2022). We observed a dose-dependent and proportional correlation between PLA and antimicrobial action, which has been reported in other studies (Valerio et al., 2016; Ning et al., 2017).

*Aggregatibacter actinomycetemcomitans* is known to form a biofilm on abiotic surfaces. The hexosamine-containing biofilm is also associated with the ability of this oral pathogen to colonize the oral cavity and is suggested to be an important virulence factor (Izano et al., 2008). In our study, we found that PLA could markedly reduce biofilm formation for 24 h, even at sub-inhibitory concentrations. A similar result was demonstrated against the biofilm of *E. faecalis* and was confirmed through confocal laser scanning electron microscopy analysis (Liu et al., 2020). In another study, Liu et al. reported that PLA inactivated planktonic and biofilm-associated *E. cloacae* cells mainly through the mechanisms of cell membrane damage and leakage of intracellular components (Liu et al., 2018). Liu et al. have shown that there were synergistic effects of PLA and slightly acid electrolyzed water that effectively inactivated *K. oxytoca* planktonic and biofilm cells (Liu et al., 2021a). The potential of PLA as an anti-biofilm compound against *P. aeruginosa* was also revealed through a detailed *in vitro* and *in vivo* intraperitoneal catheter-associated medaka fish infection model (Chatterjee et al., 2017). The present study shows that PLA might moderately disrupt the preformed biofilms of *A. actinomycetemcomitans*, although the disruption was found to be lower than 50% at all tested concentrations. Meanwhile, it has been reported that a 1–3% *w/v* concentration of PLA could effectively inhibit the development of preformed and mature biofilms of *L. monocytogenes* (Liu et al., 2017).

Furthermore, our results show that the inhibition of cell growth and biofilm formation and the breakdown of the preformed biofilm of *A. actinomycetemcomitans* by PLA were significantly higher than those by LA. Previously, a similar result was shown when a dose of 1.5% PLA was effective to curb *E. coli* O157:H7, O26:H11, O103:H2, and O121:H19 and *Salmonella typhimurium* DT104, whereas the same dose of lactic acid was ineffective (Zheng et al., 2019). Structurally, PLA has a phenyl ring in place of methyl hydrogen, which differentiates it from LA. It is suggested that this contributes to the amphiphilic nature of PLA and perhaps facilitates the interaction with bacterial membrane lipids and proteins (Rajanikar et al., 2021). In fact, several studies have pointed toward the dual mechanism of antibacterial action of PLA, namely the disruption of membrane integrity and genomic DNA stability by intercalation (Ning et al., 2017; Liu et al., 2018; Zhou et al., 2020; Fang et al., 2022). Apart from this, recently, PLA was reported to alter potassium uptake and interfere in the quorum sensing (QS) system by modulating the gene expressions of regulatory proteins or by antagonistically binding to QS receptors (Chatterjee et al., 2017; Ning et al., 2021). PLA could also inhibit the expression of virulence factors, such as pyocyanin, protease, and rhamnolipids, which are involved in the biofilm formation of *P. aeruginosa* (Chatterjee et al., 2017). In *E. faecalis*, it inhibited the exopolysaccharide synthesis involved in biofilms by downregulating the transcription of Ebp pili genes and Epa polysaccharide genes (Liu et al., 2020). In this study, we observed that PLA significantly downregulated the expression of the *pgA* gene encoding poly-N-acetylglucosamine (PGA), which could indicate its anti-biofilm formation mechanism *via* a decrease in biosynthesis of the biofilm matrix exopolysaccharide of *A. actinomycetemcomitans*. PLA markedly diminished the expressions of the two virulence-associated genes that encode major exotoxins, namely, leukotoxin and CDT. Hence, the ability of the LAB metabolite, PLA, to inhibit biofilm formation and attenuate the expression of virulence-related genes suggests its potential to impair *A. actinomycetemcomitans* colonization. The potential of postbiotics from some lactobacilli has been indicated to reduce the colonization of *A. actinomycetemcomitans* through a similar mechanism, while lactobacilli enzymes such as protease, lipase, and amylase were also responsible for mature biofilm disruption (Jaffar et al., 2016; Ishikawa et al., 2021).

The remarkable potential of PLA against *A. actinomycetemcomitans* biofilm formation and virulence gene expressions led us to investigate the production of PLA by the LAB strains *L. plantarum* MSC-C2 and *P. pentosaceus* K40, isolated in our laboratory from sugarcane and banana, respectively. *Lactiplantibacillus plantarum* MSC-C2 produced 0.8 mm PLA, a concentration about 10 times higher than that of *P. pentosaceus* K40 in MRS broth alone after 24 h of incubation. Many *L. plantarum* strains isolated from various sources have been reported to produce a considerable amount of PLA in MRS broth; however, the highest concentration was 1.38 mm, produced by *L. plantarum* IMAU10124 in 48 h without any optimizing fermentation conditions (Zhang et al., 2014). There are relatively few reports on PLA production by *P. pentosaceus*, i.e., the strain SK25 produced a high amount of 0.81 mm PLA during milk fermentation for 36 h (Yu et al., 2015); and the D-LDH from ATCC strain 25,745 produced D-PLA from phenylpyruvate (Yu et al., 2012). The conversion from PPA to PLA by LDH has been regarded as the key factor in the improvement of PLA production by LAB (Li et al., 2007; Zhang et al., 2014). In this study, we determined the PLA concentration in MRS broth with 1 mg/ml or 3 mg/ml PPA. Consequently, the PLA production was increased by many times in both strains, such that the amount produced by strain K40, i.e., 5.43 mm, surpassed that produced by strain MSC-C2. This result further validates that the initial phenylalanine conversion step to PPA is the rate-limiting step in PLA production and that this limitation can be overcome by adding PPA as the direct precursor (Lavermicocca et al., 2003; Li et al., 2007). A higher concentration of PPA has been reported to result in substrate inhibition (Zhang et al., 2014); hence, we limited the PPA supplementation to 3 mg/ml, which produced the highest PLA concentrations of about 11 mm in 24 h.

Previously, extracts from medicinal plants like *Artemisia princeps* Pampanini and *Paeonia lactiflora* Pall had been fermented with plant-derived *L. plantarum* SN13T and *L. brevis* 174A, respectively, which generated additional bioactive compounds in the plant extracts (Okamoto et al., 2020; Shakya et al., 2021). In this study, we further attempted to ferment *P. lactiflora* Pall (Paeonia Radix Alba) and *Carthamus tinctorius* extracts with PLA-producing strains MSC-C2 and K40. After the addition of 1 mg/ml PPA to the plant extracts, a PLA amount comparable to that produced in MRS broth was detected in the PR extract after 24 h of fermentation, while the CT extract produced a significantly lower amount. These PLA levels produced in MRS broth, and the PR extract were in the range of sub-inhibitory concentrations against *A. actinomycetemcomitans*. Hence, we studied the effects of PLA-containing extracts from both MRS broth and PR after fermenting with the MSC-C2 or K40 strain against *A. actinomycetemcomitans* biofilm formation. As expected, the anti-biofilm effect was proportional to the PPA concentration but inversely correlated to LA, suggesting that it was directly associated with the PLA concentration produced subsequently. These effects were cumulatively higher than the effect of PLA alone, as seen in previous results. It had been ascertained that the addition of PPA to the growth medium, resulting in the production of PLA and other metabolites that could act in a synergistic way, contributed to the improved antifungal activity of LAB strains (Valerio, 2004). Another study had reported the synergistic effect of PLA and LA against *B. cereus* by impeding several cellular processes (Ning et al., 2021). Moreover, in the presence of a relatively low LA concentration as detected in the fermented PR extracts, the anti-biofilm effect against *A. actinomycetemcomitans* was still evident after PPA supplementation. We found that fermentation indeed increased the anti-biofilm potential of the PR extract with or without the addition of PPA. The PR extract fermented by lactobacilli could produce a bioactive compound, pyrogallol, which increases the total phenolic content (TPC) and results in higher antioxidant activity (Shakya et al., 2021). Hence, the significantly higher reduction of the *A. actinomycetemcomitans* biofilm by the fermentation broth of the PR extract supplemented with PPA is plausible in the presence of a considerable amount of PLA.

Furthermore, the results showed that the fermented or unfermented PR extract could modulate the transcription profiles of the biofilm and virulence-related genes of *A. actinomycetemcomitans*. The fermented PR extract with PPA significantly downregulated all the tested target genes. While PLA alone had no effect on the expression of *dspB*, encoding dispersin B, a beta-N-acetylglucosaminidase, the fermented PR extract with or without PPA significantly downregulated it. Dispersin B is an enzyme that dissolves *A. actinomycetemcomitans* biofilms, but it can enhance the virulence of this pathogenic bacteria by mediating a “flight” response through dispersion and escape to colonize new niches when exposed to an oxidative or nutrient-limited stress-prone environment (Stacy et al., 2014; Ishikawa et al., 2021). Thus, PLA production in the PR extract *via* LAB fermentation enhanced the potential of both PLA and the PR extract to downregulate the biofilm and virulence-related genes of *A. actinomycetemcomitans*. Overall, these effects suggest a synergistic action of the PR extract and PLA produced after fermentation. Apart from the synergistic effects of PLA, LA, and other LAB metabolites, there are reports of combinational effects of PLA with other antimicrobial agents, such as bacteriocins, nisin-Z (Liu et al., 2021b), and XJS01 (Jiang et al., 2022). Hence, our results show the potential of using LAB fermentation to achieve synergistic effects of PLA and medicinal plant extracts against pathogenic biofilm formation and virulence.

## Conclusion

3-Phenyllactic acid (PLA) inhibits the biofilm formed by a periodontal pathogen, *A. actinomycetemcomitans*, and downregulates the virulence genes located on the chromosome. The plant-derived LAB strains, *L. plantarum* MSC-C2 and *P. pentosaceus* K40, produce PLA in an MRS medium supplemented with or without PPA, which was correlated with the anti-biofilm activity of their extracts. When each LAB strain was used, PLA was also produced through fermentation of the extract of a medicinal plant, Paeonia Radix Alba, supplemented with phenyl pyruvic acid (PPA). There was a combinational effect of the plant extract and PLA, produced after the fermentation process, against the pathogenic biofilm and virulence-related genes.

## Data availability statement

The original contributions presented in the study are included in the article/supplementary material, further inquiries can be directed to the corresponding author.

## Author contributions

SS and ND conceptualization and methodology. SS software, formal analysis, and writing—original draft preparation. SS, ND, MN, YI, and MS writing—review and editing. ND supervision. All authors have read and agreed to the published version of the manuscript.

## Conflict of interest

The authors declare that the research was conducted in the absence of any commercial or financial relationships that could be construed as a potential conflict of interest.

## Publisher’s note

All claims expressed in this article are solely those of the authors and do not necessarily represent those of their affiliated organizations, or those of the publisher, the editors and the reviewers. Any product that may be evaluated in this article, or claim that may be made by its manufacturer, is not guaranteed or endorsed by the publisher.
